# Detecting the provenance of price hike in agri-food supply chain using private Ethereum blockchain network

**DOI:** 10.1016/j.heliyon.2024.e30972

**Published:** 2024-05-21

**Authors:** Maksuda Haider Sayma, Md Rakib Hasan, Mahmuda Khatun, Alimul Rajee, Amena Begum

**Affiliations:** a Dept. of Information and Communication Technology, Comilla University, Cumilla, Bangladesh; b Dept. of Computer Science & Engineering, Comilla University, Cumilla, Bangladesh

**Keywords:** Price-hike, Blockchain, Ethereum, Smart contract, Agri-food, Supply chain, IPFS, Decentralized application, Transparent, Traceable

## Abstract

The rise in the cost of essentials affects every nation around the world, but it has become a major concern for developing nations. It is getting increasingly difficult to keep up with rising prices for everyday items in these countries, where the majority of the population is from the middle class or lower middle class. Inflation, pandemics, wars, and other important variables all contribute to price increases. There may be another significant factor at play, which is supply-chain corruption. The supply chain's unreliable, chaotic, and opaque nature is to blame for this corruption. We are concentrating on the agri-food supply chain in our study. Because many of the current agri-food supply chains are intricate and challenging to monitor, dishonest parties can exploit the situation. Therefore, we suggested a thorough blockchain-based agri-food supply chain to identify the source of price increases. The private Ethereum blockchain was used in the suggested system. Since the private Ethereum blockchain is more efficient, safe, and fast, it was chosen. Smart contracts were created to describe the system and its underlying rules and laws. Furthermore, in order to showcase the usefulness of our smart contracts, we exhibited a sample decentralized application to support our hypothesis. We also gave the system a complete security and vulnerability assessment to make sure it is operating properly and is protected from threats and attacks. Due to the use of blockchain, the system is immutable, transparent, and simple to track and monitor. The proposed system has demonstrated greater transparency, traceability, reliability, speed, security, and cost-efficiency compared to conventional systems. It effectively traces the origin of corruption in the supply chain, providing a more straightforward means to tackle concerns related to price hikes.

## 1 Introduction

The price hike is a universal challenge that significantly affects the day-to-day life of the common people. There are various reasons like inflation, pandemics, wars, supply constraints, accelerated population expansion, economic growth, deficit spending for development, rising money supply, insufficient industrial and agricultural output, and expensive imports that can cause price hikes. Unreasonable price increases have an influence on the global economy in addition to the lives of ordinary people. A substantial price hike may lead to a decline in social stature. Even while it affects the entire nation, the poor and lower middle class are the ones who have been bearing the brunt of it. Food prices have soared across the world as a consequence of rising costs for essential commodities in the global market. Developing countries are also affected by the horrible damage inflicted by the globality of price hikes. The majority of people had to deal with the dreadful consequences of the global “lockdown,” which resulted in job losses and a sharp decline in the standard of living, even though these countries were able to survive the epidemic. The lockdown measures were anticipated to produce a disturbance in the food supply chain that raised food costs, which exacerbated the issue of food insecurity [1]. The main consequence of COVID-19 has been a linear rise in global food prices since February 2020. The international price of food commodities peaked in September 2020 at 97.19 points, according to the FAO Food Price Index (FFPI). The key reasons for the price increase during COVID-19 were high demand, panic buying, overstocking of items, a labour shortage, the shutdown of food processing plants, and interruptions in the global supply chain owing to travel restrictions. The COVID-19 pandemic may also have long-term effects that include insufficiency, which can result in unemployment, excessive costs, and reduced production [2]. Over the past six months, the prices of almost all commodities have climbed dramatically, placing a further economic burden on the middle and lower-income classes and elevating the price increase to the position of global concern. This excessive price increase has the potential to harm society's social-economic dynamic by increasing the crime rate. Famine in developing nations may result from an inability to regulate the price increase. Although the aforementioned factors are to blame for the price increase, there is another significant factor that contributed to the current state of affairs, and that is supply chain corruption. In order to build a sustainable supply chain, corruption can be named as an important yet poorly understood obstruction. Although supply chains face a significant risk from corruption, especially in emerging economies, it has become a relevant issue everywhere and for every single supply chain across the world. In some cases, stakeholder collaboration may increase the risk of corruption in supply chains [3]. The governments have been working hard to pinpoint and address this problem, but a complex and untraceable supply chain structure has always stood in the way. The essential calculations, such as transportation costs and overall expenses, could be easily changed when a product moves through different supply chain participants (manufacturer, wholesalers, retailers, etc.), facilitating bribery and corruption, which ultimately leads to inflation. Such actions put the country's entire economy in jeopardy [4]. For example, S.A. Sabur et al. found that the fundamental reasons for the price increase were not Bangladesh's poor production and high consumption of potatoes but rather a fake crisis that was fabricated by a few profit-seeking traders [5]. In their paper, Mohammad Kamrul Hasan et al. suggested a blockchain-based mechanism for tracking pricing increases in industrial enterprises. In addition to inflation, they believed that price increases were primarily the result of bribery and corruption between buyers and sellers [6]. There are always going to be middlemen in the supply chain from the supplier to the consumer. These middlemen and the non-structural, opaque supply chain can occasionally raise prices while depriving both the producer and the customer. This is the type of problem we are attempting to address by suggesting a blockchain-based supply chain. The mass population struggles with the exorbitant cost of daily necessities. Although there are several problems associated with this issue, one of them may and should be resolved by offering a transparent and traceable supply chain. Given all the information, now is the ideal time to put such structures in place. Although blockchain gained popularity when it was used to host popular cryptocurrencies, its application is much broader than this. Due to its immutability quality, it may be advantageous to utilize it in systems that call for tracking and tracing in order to prevent various socio-economic problems. A distributed, or decentralized, ledger known as a blockchain is a digital platform for securely and immutably maintaining transactions between many individuals [7]. Additionally, the ledger itself can be set up to automatically initiate transactions. The primary goal of blockchain is to allow a large number of anonymous participants to conduct confidential and verifiable transactions with one another in cryptocurrency networks, which were designed to replace fiat currency. In the case of the supply chain, the goal is to provide a straightforward yet accountable and transparent system where stakeholders may conduct transactions in a secure manner. For security reasons and because they make it easier to monitor and trace transactions, permissioned blockchain solutions are crucial for supply chains. Vitalik Buterin proposed Ethereum, an open-source, decentralized platform for smart contracts and decentralized applications, in 2014 [8]. Ethereum underwent the eagerly awaited “Merge” transformation on September 15, 2022, switching its consensus algorithm from proof-of- work (PoW) to proof-of-stake (PoS). The outcome was a 0.2 % decrease in global electricity consumption and a 99.95 % reduction in Ethereum's energy consumption rate. It hosts ether, a cryptocurrency with a market capitalization that ranks second only to bitcoin and was first introduced on July 30, 2015. Along with the native token, ERC-20, ERC-721, ERC- 777, ERC-1155, ERC-1155, and ERC-4626 tokens are also supported. It is widely used because of its decentralized structure and emphasis on smart contracts.

The system described in this paper, which uses a private Ethereum blockchain network, can revolutionize the current agri-food (Agriculture and Food) supply chain and provide a clear concept about the cause of price increases in the agri-food supply chain. We suggest a broad system that can be used across a wide range of goods and industries. The proposed system can establish a secure, traceable, and transparent agri-food supply chain. It enables us to trace the provenance of a product and ensure that each stakeholder receives a fair share. Our primary objective is to identify the source of illegal price increases and the responsible parties within the agri-food supply chain. The study also indicates its potential to prevent or mitigate price hikes, as discussed in the outcomes of the use case analysis. By identifying and addressing root issues within the agricultural supply chain, the study aims to eliminate problems, thereby contributing to a decrease in unwarranted price escalations.

The main contributions of the proposed system are as follows.

• We proposed a through-and-through agri-food supply chain system based on blockchain.
• The technology will allow the general public and the government to view the necessary product information. Additionally, the system is faster, more secure, and traceable while utilizing the private Ethereum blockchain.
• The interactions between stakeholders were recorded using smart contracts.
• We proposed to make use of the Proof of Authority (PoA) approach since it uses less processing power and simpler hardware.
• The system can keep track of every transaction, which makes it possible to identify the source of corruption.
• We used the InterPlanetary File System (IPFS) for storing data [9].
• To demonstrate how the system functions, we also created a demo dApp (decentralized Application).
• The system has been examined and verified to be safe from typical vulnerabilities and threats. We also included the costs for each contract in terms of gas and fiat currency.
• Additionally, this system can be utilized to assure food safety and minimize food waste.

This research provides cutting-edge background knowledge on the advantages of a blockchain-based agri-food supply chain over a conventional centralized framework. Section II includes recent work on blockchain-based agri-food supply chains, along with a few cold chains. A succinct analysis of why blockchain-based solutions are superior to centralized ones is also provided. It also provides some idea about the scope and challenges of the study. Section III provides a description of how the whole research was conducted. The requirements and the procedure of the study are explained in this section. Section IV presents the experimental results, provides an illustrative analysis and cross-checks the results. In this section, the evaluation of the performance of models is done along with a discussion. Section V summarizes and concludes the study. It also provides the limitations of the study, recommendations and future directions. The suggestions were based on the findings of the study and were intended to address some of the concerns that emerged during the process.

## 2 Literature review

The studies that served as the basis for the research and the studies that are analogous to ours are addressed and reviewed in this section. In recent years, researchers have used blockchain technology to address inefficiencies in the agri-food supply chain, and blockchain has proved effective in doing so. We made an effort to provide a system that could detect the source of price increases in the agri-food supply chain, despite the fact that all of these systems and frameworks aim to transform the food business by making the supply chain more transparent and dependable.

In 2022, Dayana D. S. and Kalpana G. proposed a technique that develops a food production system based on the Ethereum blockchain that lowers transmission costs, boosts efficiency, and boosts security throughout the food supply chain [4]. Agri-Insurance is a novel component of the proposed system that is in charge of offering insurance to farmers for the purchase of seeds and supplying prompt compensation to the farmers during natural calamities. Every transaction is documented in a distributed shared ledger connected to IPFS. The authors also proposed that when crops and goods are delivered, smart contracts use cryptocurrency to automatically pay all stakeholders. Yakubu BM et al. designed a decentralized system for a safe and traceable rice supply chain, that is named RiceChain [10]. In order to minimize contamination and enhance rice product safety, the suggested framework was supposed to track and monitor all contacts and transactions between all participants in the rice chain environment through smart contracts and Ethereum Blockchain. The suggested approach also included a method for collecting customer reviews, allowing all stakeholders to access the most recent data on product quality and make improved supply chain recommendations. The system made use of IPFS with information about rice production. An entirely blockchain-based verifiable agri-food supply chain was proposed by Angelo Marchese et al., along with a prototype implementation [11]. The suggested method encourages supply chain automation using smart contracts, which makes it more unambiguous, uncompromising, and secure. They used the Hyperledger Fabric permissioned blockchain. The research's goal was to find a scalable and practical replacement for the current centralized system that would reduce fraud and increase customer satisfaction. ShrimpChain is a blockchain-based, smart contract-empowered, and accumulative score-based certification conceptual model that Md Akhtaruzzaman Khan et al. proposed to address the socio-technical concerns pertaining to shrimp farming and the supply chain in Bangladesh [12]. The proposed work aimed to assist shrimp farmers by creating a comprehensive and verifiable sys-tem. It also aimed to assist the government in taking effective measures at the shrimp export facility. The suggested solution combines Corda and Ethereum to create a hybrid private-public blockchain network. The hashes of the data stored in IPFS will be accessible on the public (Ethereum) layer. To guarantee the integrity of information and food security, Bawankar Chetan Daulatrao et al. proposed a complete strategy for the Agri-Food supply chain using Ethereum and smart contracts [13]. In contrast to conventional centralized systems, they aimed to create a transparent system that would prevent fraudulent and malevolent behaviour. IPFS was utilized to store pertinent data as well as for security reasons. Along with simulations and assessments of smart contracts, this study's findings also contain evaluations of security and susceptibility. A permissioned blockchain paradigm employing the Ethereum blockchain and smart contracts (coded using Solidity) has been proposed by Ibtisam Ehsan et al. [14]. By offering a distributed solution, the system aimed to prevent the limitations caused by centralized systems' lack of traceability and ambiguity, provenance records, transaction history, and food quality certification, thus boosting consumer experience and trust. The IPFS and PoA consensus algorithms are employed in the system to increase its security and dependability. By efficiently creating generalized Ethereum-based smart contracts that automate the process to cut down on development time and guarantee the dependability of the contracts, Lodovica Marchesi et al. came up with a unique method to boost the reusability of code and modules [15]. By automatically generating the smart contracts and the user interfaces required to communicate with them to administrate the system from the start of a product's life cycle to its conclusion, they were able to construct a system that functions semi-automatically. They also used a novel case study (the production of honey) to demonstrate how the framework functions. Paulo R.V. de Carvalho et al. proposed an optimized BCT-based supply chain model, taking deployment expenses and profits into consideration [16]. They discussed ideas including quality-based product differentiation, data-enabled products, and using supply chain information for commercial gain. They used blockchain to confirm data on transit schedules. The BCT supply chain concepts were demonstrated using the global fresh-cut flower supply chain.

By suggesting a search for the issue of mismatches that occur along the supply chain from upstream to downstream, Ratna Ekawati et al. proposed the design of a unified white crystal sugar agro-industrial supply chain system based on blockchain technology in order to increase competitiveness in achieving food security and resilience in 2021 [17]. The use of a decentralized blockchain consortium with smart contracts on the Ethereum platform was recommended by the authors.

To increase supply chain visibility, Arwa Mukhtar et al. proposed a blockchain-based supply chain network concept in 2020. Information sharing, traceability, and inventory visibility are the three visibility measurement qualities that the model focuses on improving [18]. The suggested approach includes platforms for data sharing, traceability, and inventory visibility built on top of blockchain technology. The model is a development of the Hyperledger Composer Supply Chain Network (HCSC), which is built on the Hyperledger platform.

In 2019, Kok Yong Chan et al. released “Prochain,” a concept for a permissioned blockchain-based industry [19]. “Pepper” is used in this study as an agri-food domain. With Sawtooth, the framework achieved complete transparency and traceability, whereas Fabric is a little bit more difficult.

Researchers used both permissioned and permissionless blockchains for supply chain solutions. The majority of academics favour Ethereum or Hyperledger over alternative blockchain technologies because both of these products have shown promising performance. While Hyperledger (permissioned) is preferred for business frameworks where confidentiality is crucial, if necessary, Hyperledger allows transactions between two parties to take place without the involvement of a third party. Additionally, as Hyperledger lacks a native currency, transferring digital assets is not an option. Even though all of these attributes may be suitable for some organizations and enterprises, they are not for ours. The choice of the best approach is entirely dependent on the issue at hand. In this case, our goal is to create a system that is completely *trans*parent and visible to every participant in the system. This, in our opinion, is the most effective strategy to identify and stop systemic illegal price hikes. Both private and public Ethereum are possible while maintaining transparency in both cases. As it has its own native currency, it has the potential to handle digital assets via smart contracts. Additionally, Ethereum allows the development of decentralized apps more effectively than other blockchain systems. Additionally, because access is restricted to the individuals who require it, private Ethereum networks are faster and more secure than public ones. But unlike other alternatives, Ethereum consensus is applicable even in private settings.

## 3 Research Methodology

This section goes through the step-by-step procedures needed to develop such a system. Here, the necessary software tools are listed. From implementation to testing, all aspects of the development process are explained. First of all, we proposed a much simpler and more transparent agri-food supply chain instead of the complex and non-transparent one currently existing. Then we designed the proposed end-to-end (farmer-to-consumer) agri-food supply chain based on a private Ethereum blockchain using smart contracts. The designed algorithms are explained along with their implementation. We also made use of InterPlanatary File System (IPFS) to store data and the hash of that data was stored on the blockchain. Finally, a simple demo of the system was implemented. The smart contracts were programmed using solidity. For the development framework, truffle was used. Ganache was used as our local blockchain. To communicate with the Ethereum blockchain environment, we used the metamask wallet. The system was first tested on Remix IDE then was shifted to Visual Studio code for making a dApp (decentralized Application), where working on UI was easier.

### 3.1 Conceptual framework

A government official or officials will be included in the admin panel that will be constructed first. For instance, local agriculture officers are present in each district of Bangladesh and work closely with the area's farmers and traders. The administrator can provide the local farmers and merchants with instructions on how to connect to the local blockchain. The supply chain can be monitored by additional government personnel, such as consumer rights agents, who can also investigate any anomalies or complaints. Each stakeholder must be added to the system by the administrator (local government official). The administrator must approve each entity. The farmers should use the dApp to add their products and essential data to the system. Detailed information must be added to IPFS and the hash link for these details must be published together with other information in the system. This will cause a product availability event to occur. Then a series of exchanges take place amongst the parties involved. We recommended using the Proof of Authority (PoA) consensus algorithm since it validates transactions more quickly and reliably by leveraging a user's identity or reputation. Each transaction is added to the blockchain when it has been validated. The IPFS system will contain all transactional information. Farmers, distributors, retailers, and consumers were all connected to the system in the demo dApp, but for a large-scale application, the customers may receive the necessary information by QR code. Every operation in the decentralized application follows the implied rules of the smart contracts. Anytime a participant tries to break the rules, the transactions will immediately stop processing. Additionally, since each member has a copy of the ledger, any changes to the records may be quickly traced back to their inception.

**Fig. 1:**
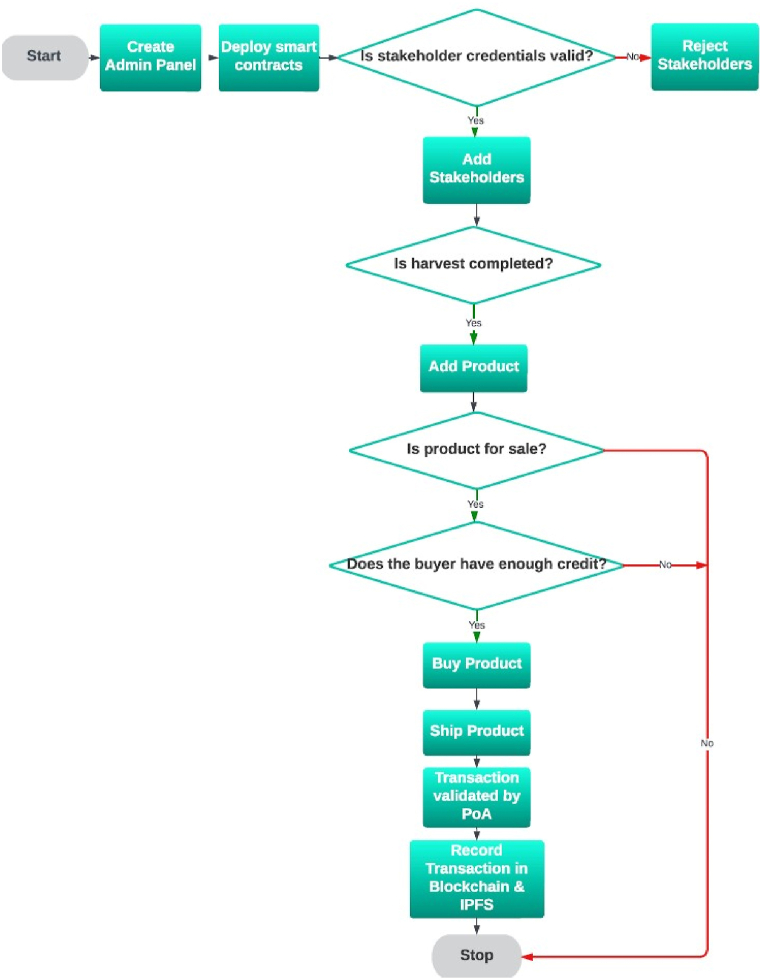
Flow chart of the whole procedure.

The flowchart depicted in Fig. 1 illustrates the fundamental operations of the system in a straightforward manner.

### 3.2 System design & implementation

**Fig. 2:**
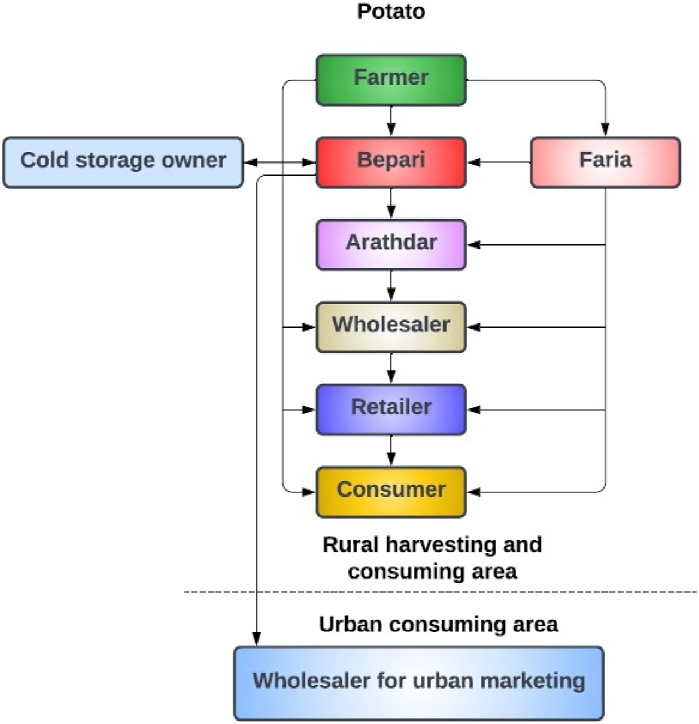
Supply chain for potatoes in the study area.

**Fig. 3:**
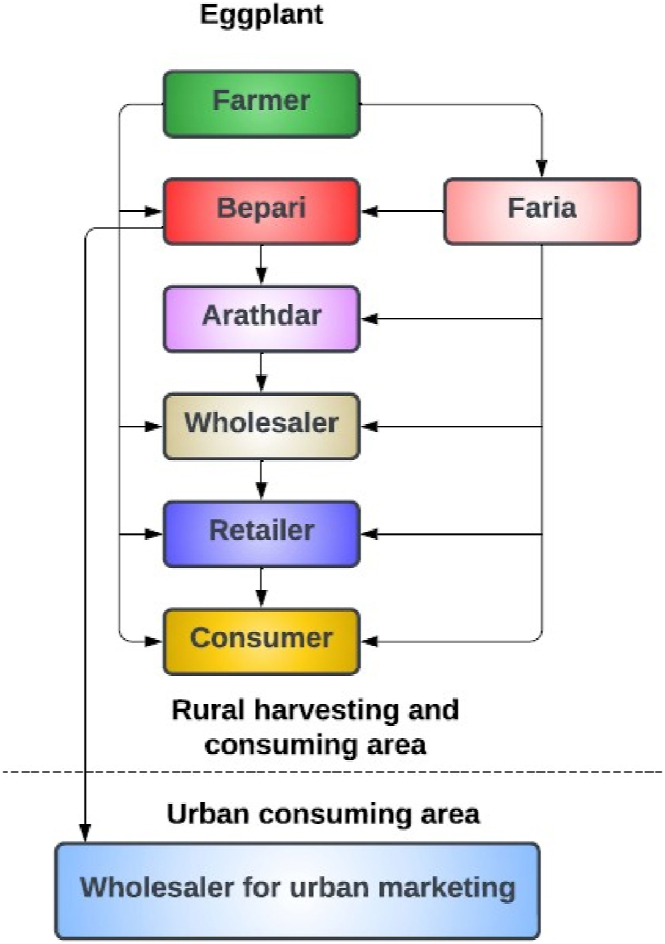
Supply chain for eggplants in the study area.

In order to explain the issue in question and demonstrate the effectiveness of our solution, we talked about Bangladesh's agri-food supply chain, which may be similar to the ones that are available in many other developing countries. Fig. 2, Fig. 3 show two supply chains for vegetables (potato and eggplant) in the Lalmonirhat district of Bangladesh provided in the research done by Utsarika Singha et al. [20]. It is evident from Fig. 2, Fig. 3 that the supply chains involve far too many intermediaries. Because Bangladesh's traditional agri-food supply chains are so intricate and difficult to track, dishonest individuals can more easily act illegally to fulfil their wants while avoiding detection. Furthermore, it is apparent that chains such as these make it difficult to track transactions, making it practically impossible to determine where fraud originated.

Even though there are regional variations, these characteristics can be seen in nearly all supply chain networks. For instance, the local rice farmers, vegetable growers, and a scientific official from the Bangladesh Rice Research Institute- BRRI in Cumilla, Bangladesh all claimed to have a supply chain that was somewhat distinct from this one, but the basic structure was the same and the system was neither stable nor transparent. In conclusion, unnecessary middlemen and non-structural transactions constitute the existing agri-food supply chain. So, we suggest a far more straightforward, general, and manageable agri-food supply chain.

**Fig. 4:**
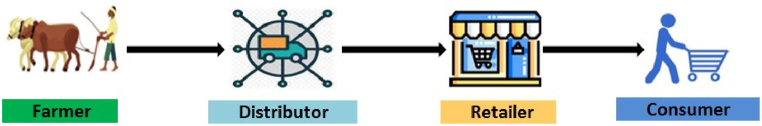
Proposed agri-food supply chain.

**Fig. 5:**
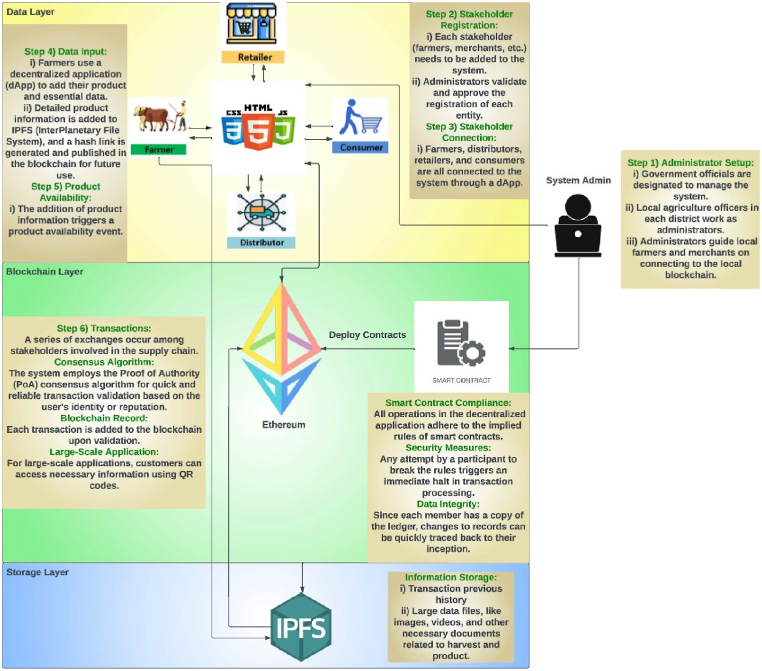
System model of the proposed agri-food supply chain.

Fig. 4 shows a simple agri-food supply chain, which is the simplified version of Fig. 2, Fig. 3. This type of chain can be easily implemented and maintained. The complete blockchain-based end-to-end system with IPFS storage is depicted in Fig. 5. The system admin and every stakeholder are in constant communication with the system. The system admin adds each participant to the system and deploys the smart contracts to the private Ethereum blockchain. When a member is no longer relevant, only the admin has the authority to remove them. Each member must have an Ethereum account before they may be added to the system with proper authentication. The local Agriculture Officer, who is far better connected to the local farmers, may serve as the admin in a real-world application.

The layers from Fig. 5 are explained below:

**Data Layer**: As seen in the above figure, farmers, distributors, retailers, and consumers are the actors or participants who contribute to the system. Every interaction they have is considered a separate transaction in the system. They all own a copy of the system's current state after the admin has added them to it. Additionally, every stakeholder involved in the system must consent to any changes made before they may be included in a block. Smart contracts are used to record every transaction and add them to the chain.

**Blockchain Layer**: The relevant contracts define every aspect of adding, deleting or authorizing a member. The supply chain contract contains information that explains the transactions between these parties. These contracts are all written with Solidity. Since we are building the system on a private blockchain network, we are proposing Proof of Authority (PoA) as the consensus mechanism in this case. In this technique, a member's reputation (which is granted via authorization) is sufficient to validate a transaction without the need for actual mining. This technique is quicker, safer, requires less sophisticated hardware, and is resistant to attacks like the 51 % Attack and Denial-of-Service (DoS). A corresponding event is generated and logged in the logs each time a transaction takes place. All the transactions are validated by the authorized members according to the PoA consensus. All transactions are added to a block, time-stamped, and have their members validate them.

**Storage Layer**: We utilized it to store pertinent data from the blockchain to the InterPlanetary File System (IPFS) instead of conventional databases. A peer-to-peer (p2p) storage network system known as IPFS is used to store and access information, documents, web pages, and applications. To retrieve the stored data, a Container Identifier (CID) or hash is provided [9]. Instead of storing the actual data, which could be resource-intensive, these hashes are kept in the chain. In addition, the farmers add all the detailed information about harvesting to the IPFS, and if any members require it, a hash is provided in the chain.

Algorithm 1 is implemented in four of the contracts in the addFarmer(address account), addDistributor(address account), addRetailer(address account), and addConsumer(address account) functions. All of these functions are only accessible by the admin.

**Figure:**
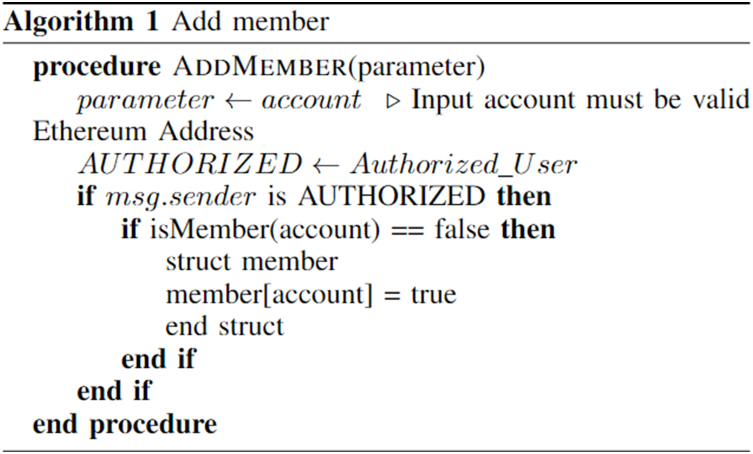

A member can be added or removed only by the admin. The SupplyChain contract inherits the Farmer, Distributor, Retailer, and Consumer contracts. If anyone other than the admin tries to perform any of the above-mentioned functions, the transactions will not go through, throwing an error. Emitting events will be kept as records in the log. Instead of just saving to public storage variables in our smart contracts, events enable us to “publish” information on the blockchain in a way that is more accessible and gas-efficient [21]. Next, the item/product should be added to the chain. This function can only be called by the farmer. Here the addresses are the Ethereum addresses of the members. Stock Keeping Unit (SKU) and Universal Product Code (UPC) are combined to create the product code. Here, a product is uploaded to the system together with the essential details, such as the farmer's location, the farm's location and condition, the growing conditions of the product, the next link in the chain, and the product's pricing. The “Harvested” event is triggered, informing the stakeholders that the product is now available. Algorithm 2 shows the selling of the product from the farmer to the distributor. In this case, the entity that wants to buy the product has to be an authorized member of the network.

**Figure:**
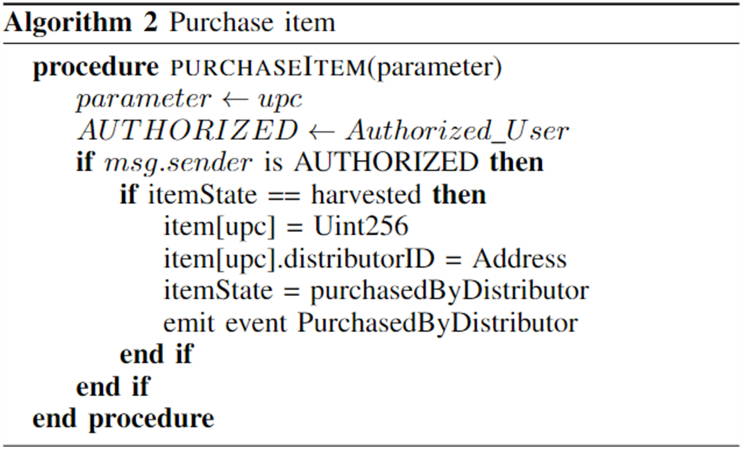

Algorithm 3 shows the shipping of products from a farmer to a distributor. This function can only be called by the corresponding farmer and only when the product is sold to the distributor. Then the state of the product changes from “PurchasedByDistributor” to “ShippedByFarmer”.

**Figure:**
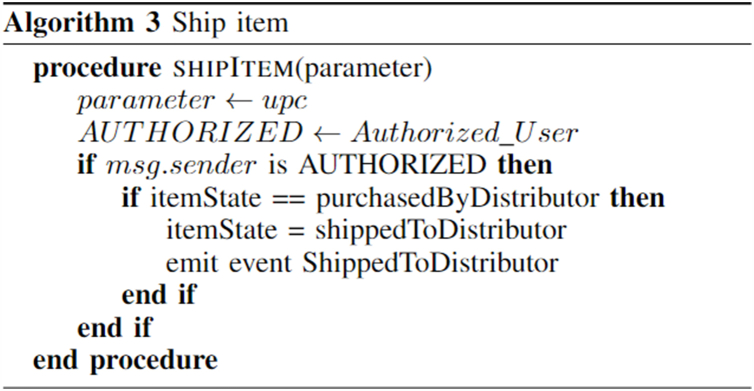

**Figure:**
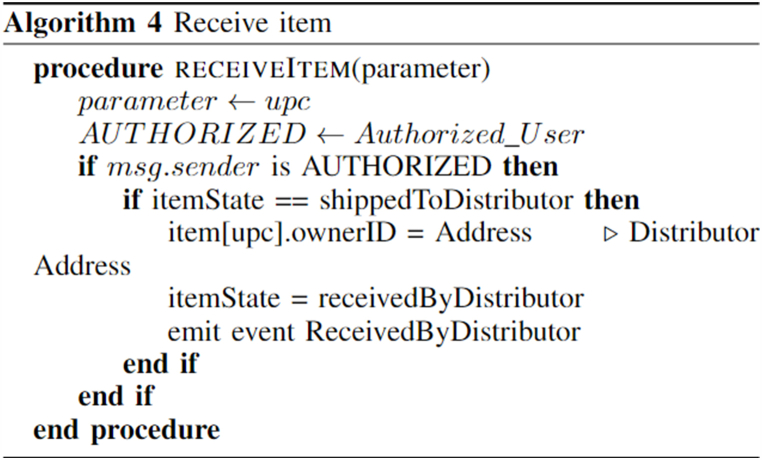

Algorithm 4 shows that the distributor confirms that he/she has received the product and the ownership of the product now changes from farmer to distributor. Also, the state of the product changes from “ShippedByFarmer” to “Received- ByDistributor”. Algorithms 2, 3, and 4 are also applied to the appropriate events like buying, shipping, and receiving of products by retailer and customer.

### 3.3 Implementation requirements

**Fig. 6:**
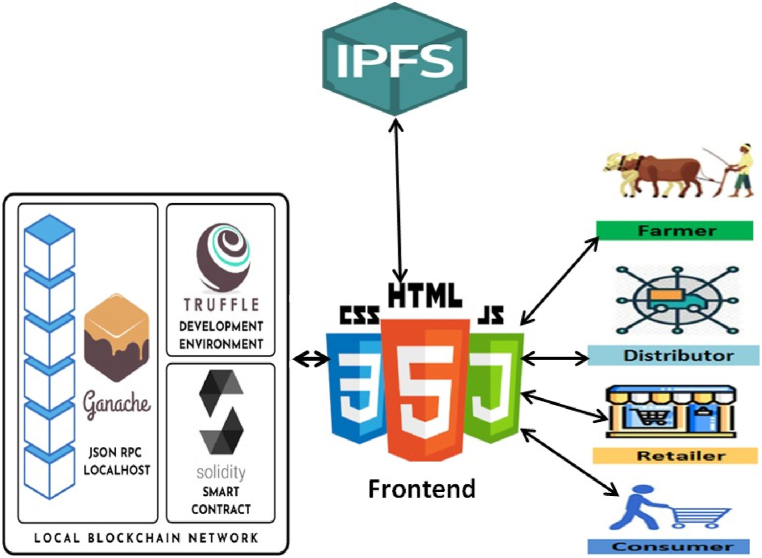
Architecture diagram of the decentralized application.

We utilized the truffle framework to build the supply chain dApp and ganache for the local blockchain. Basic HTML, CSS, and JavaScript were used for user interfaces. The user interface demonstrates the basic functionalities of the system. It serves as a demo app to demonstrate how well the system functions. Truffle is used to develop, migrate, and deploy Solidity smart contracts to the local blockchain. The frontend connects to the local blockchain and smart contracts using web3.js. Users can connect with the system and conduct transactions using their metamask wallet and their public address. The dApp's architecture diagram is displayed in Fig. 6. Data is stored using IPFS, and a blockchain is utilized to store the data hash.

**Fig. 7:**
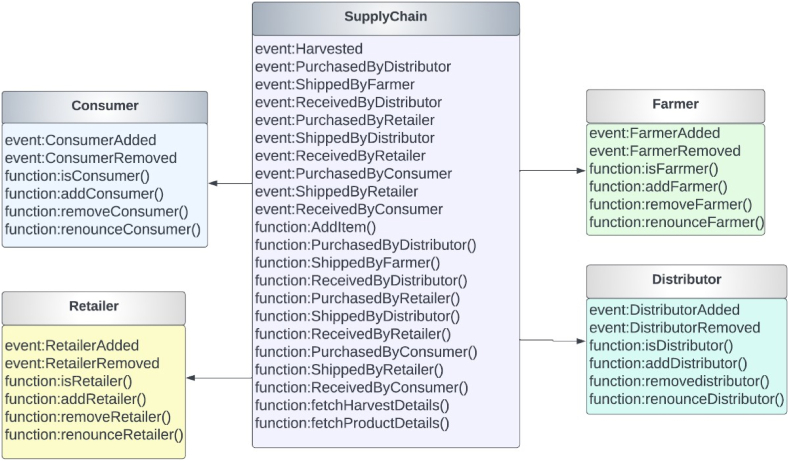
Smart Contract functions and events.

**Fig. 8:**
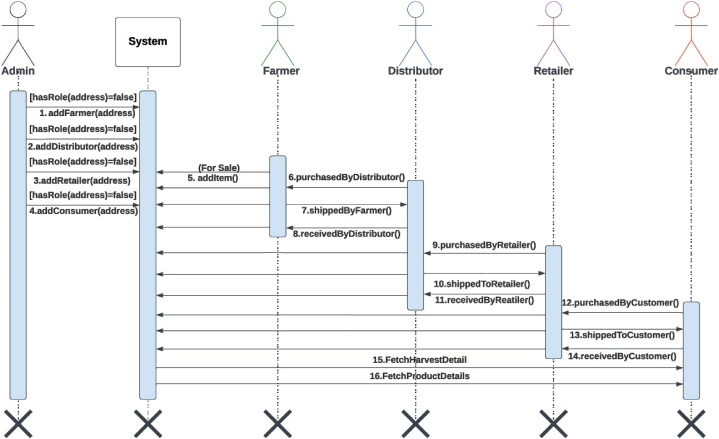
Sequence diagram.

To direct how the interactions between the stakeholders should go, we applied the aforementioned algorithms to the smart contracts. We will now go into greater detail on the design of smart contracts. Fig. 7 shows the events and functions generated in the smart contracts. The contracts Farmer, Distributor, Retailer and Consumer implemented the basic structure from the Roles library and then each of these contracts was inherited by the SupplyChain contract. Fig. 7 shows the smart contract functionalities and the associated events that are generated during each transaction. Only those with the proper credentials and when the item has moved past a specific state are able to use any of these functionalities. Fig. 8 shows the sequential operations of the functions from the smart contracts.

## 4 Result and analysis

In this section, we present the findings of our research and provide an analysis to support our assertions. We also performed a thorough examination of security vulnerabilities to demonstrate that the system securely performs its intended functions. A cost analysis of the smart contracts was also presented. Three categories—security analysis, cost analysis, and vulnerability analysis—were used to discuss the findings. The parameters of the experiments, including transparency, traceability, reliability, cost, and security, are described in detail within these categories.

### 4.1 Security analysis

Authenticity: In private chains, every entity that interacts with the system in some way must be authenticated first. Additionally, only stakeholders who have been pre-approved can activate any functionality. It increases the system's dependability and security. This type of system is also immune to man-in-the-middle attacks.

Transparency: Since all system transactions are visible to all parties involved, it is more difficult for a single entity to commit a malicious act. Each event is recorded in the log and given a time stamp. Therefore, it is simple to trace and track every transaction to its source, meeting our main objective of locating the tainted entity that squanders resources for private gain.

Traceability: Blockchain technology offers a potential solution for enabling traceability with a secure and immutable information trail [22]. Within the blockchain, transparency is a fundamental characteristic. Every transaction is visible to all network participants, as it is stored in blocks in a sequential chain, with each member possessing a complete record of these transactions. This means that any involved party can access the full transaction history associated with a specific Ethereum address. In a blockchain network, each participant is equipped with a unique cryptographic identifier in the form of public and private keys. These identifiers play a crucial role in verifying and signing transactions. Once a transaction is integrated into the blockchain, it becomes immutable, safeguarding the integrity of the transaction history. Any attempts at fraudulent or unauthorized transactions are automatically rejected by the network, as all pre-approved participants must validate these transactions. Furthermore, each block within the blockchain is marked with a timestamp, indicating when the transactions took place. This chronological order is essential for facilitating traceability.

Reliability: A blockchain-based system is highly regarded for its reliability due to several key attributes. It offers transparency, ensuring that every transaction is visible to all network participants. Transactions are also traceable, making it possible to follow their history. Importantly, blockchain is inherently resistant to fraud. This resistance arises from the fact that each stakeholder maintains a copy of the entire ledger, ensuring that no single party can manipulate the data. Furthermore, each transaction is meticulously recorded with chronological order, timestamps, and cryptographic hashing, enhancing its security and trustworthiness.

Credibility: A reliable system is required to fend off attempts like the replay attack. The entities must be certain that their interactions with one another are trustworthy and honest in order to create a credible system. Every stakeholder is registered, and the interactions between the entities are immutable in the proposed system, giving the system credibility.

Autonomy: The PoA consensus mechanism is appropriate for the suggested system. Here, all the transactions are validated by the pre-approved stakeholders, making it safer from unauthorized and malevolent parties. A 51 % attack can be avoided via an autonomous system with authenticated members.

Auditability: Once implemented, smart contracts cannot be changed. No interaction in the system can be altered unless all system entities agree to do so (which is highly unlikely). Because of this, the entire system is thoroughly audited before deployment to ensure that there are no defects or security flaws.

Non-repudiation: Each participant has a separate public and private address. Their public addresses, as well as each *trans*action they perform, are recorded on the blockchain. No party can arbitrarily withdraw from an agreement.

Accountability: We just included one admin in the suggested system; additional ones could be added if needed. The admin must report any suspicious or malicious activity in the system, and so must the other stakeholders. Here, we're presuming that the admin or regulator is sincere and impartial. The system data is tamper-proof, so even if the admin were to act arbitrarily, other stakeholders might object and take appropriate action.

### 4.2 Cost analysis

The term “gas” denotes the metric used to express the amount of computational power necessary to carry out particular activities on the Ethereum network. Each Ethereum transaction has a cost since they all need computing resources to complete. The charge needed to complete a transaction on Ethereum is referred to as “gas” [23]. Our “SupplyChain” smart contract is the one with the most gas consumption as all the functions are executed in this contract. Still, this value is easily accepted as minimal. The other contracts have the same functionalities, resulting in the same gas consumption. (See Table 1)

**Table 1:** Gas consumption of the smart contracts.

Smart Contracts	Deployment Gas	Cost in Ether	USD
Farmer	306084	0.00612168	$9.43
Distributor	306072	0.00612144	$9.43
Retailer	306072	0.00612144	$9.43
Consumer	306084	0.00612168	$9.43
Supply Chain	2321137	0.04642274	$71.50

Table 1 displays every contract as well as the corre-sponding gas, the cost in Ether, and its equivalent value in fiat currency. The total cost in USD is $109.22. While 1 Ether = 1540.18 USD [24].

**Fig. 9:**
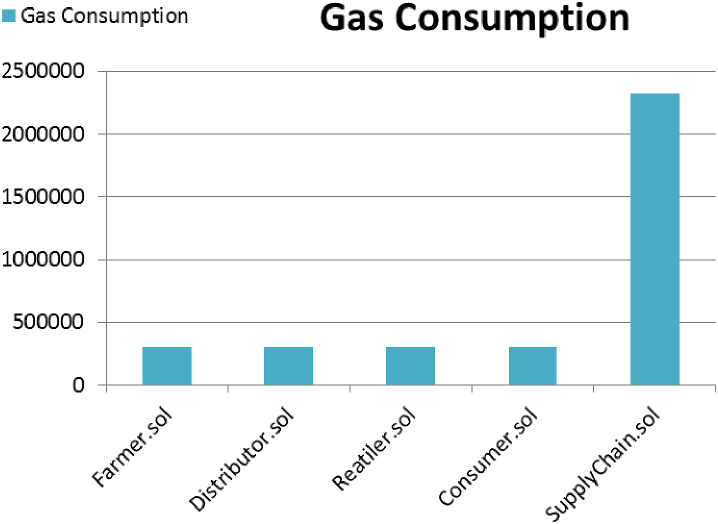
Visualization of gas consumption of smart contracts.

Fig. 9 compares the gas consumption of the various con-tracts. The supply chain contract inherits all other contracts, and because all functions are triggered by this contract, its gas consumption is higher than that of the others. These prices are shown according to the public Ethereum network. Transaction expenses (gas fees) are lower compared to the public Ethereum network and can be adjusted to specific needs. This cost is substantially less than the financial losses incurred due to corruption in the current system.

### 4.3 Vulnerability analysis

Smart contracts must be protected from obvious threats since they cannot be altered after they have been deployed. Therefore, it is crucial to make smart contracts as secure as possible from known attacks. Otherwise, the integrity of the system could be compromised. We conducted an analysis of the smart contracts using Oyente [25]. Oyente is used to identify possible weaknesses in smart contracts.

The reports are provided below.

According to Table 2, smart contracts are secure from well-known vulnerabilities. The proposed system was found safe from parity multisig bugs, call stack depth limitation, transaction ordering, timestamp dependency and re-entrancy attacks [26].

**Table 2:** Vulnerability analysis report of the smart contracts (Farmer.sol, Distributor.sol, Retailer.sol, Consumer.sol, SupplyChain.sol.

Parameters	Result
Parity Multisig Bug 2	False
Callstack Depth Attack Vulnerability	False
Transaction-Ordering Dependence	False
Timestamp Dependency	False
Re-Entrancy Vulnerability	False

### 4.4 Proposed system in food supply chain

Uncertainty regarding food's provenance, food contamination, food fraud, locating the source of tampering or adulteration, and food waste are the major food-related difficulties that affect everyone. By effectively utilizing blockchain technology, all of these problems might be resolved. The farmers who cultivate our food are where the proposed system begins. Location, the altitude at which the plants are grown, irrigation practices, plant growth conditions like soil, temperature, humidity, fertilizer, and pesticide use, as well as other information like the date of harvesting and dispatching to the next link in the chain, are all required to be stored on the blockchain. They must also present any necessary credentials to demonstrate that the food they have grown meets certain criteria, such as being organic or free of animal products. Information about the product's health and other details like the storage setup, inventory, temperature, etc. must be gathered as it passes up the chain to other stakeholders. Internet of Things (IoT) devices can also be used to gather data and confirm the information that participants have provided. This allows for the tracking and resolution of any significant food-related issues. The participants can better understand where to invest by using this kind of transparent system that gives a clear picture of supply and demand for a certain commodity. This reduces waste.

This system is adept at eliminating unnecessary delays within the supply chain, ensuring the swift delivery of products to the market. We have specifically designed this system for the agri-food supply chain, where it is critical to make products available in the market soon after harvesting, as they are highly perishable. In this system, products are recorded from the moment they are planted and are tracked through every stage of their lifecycle, providing all stakeholders with valuable insights into product availability, quality, and pricing. This information empowers stakeholders to make more informed decisions, ultimately influencing supply dynamics. Furthermore, this technology fosters better coordination among producers by offering a shared platform for monitoring the entire product lifecycle and responding to shifts in demand. Producers can adjust their production based on market demand data derived from the blockchain, thus reducing the risk of oversupply or shortages.

Our research has revealed that food price increases in Bangladesh are predominantly attributed to the actions of unscrupulous business individuals who artificially inflate prices. The problem, as highlighted in our examination of a practical case, stems from the lack of substantial proof to support price increases. The proposed system offers a remedy through enhanced transparency, as Ethereum allows every stakeholder to access the complete transaction history. Under this system, the government would be responsible for establishing standardized pricing for various agricultural products. This pricing would be determined based on comprehensive inputs, including data provided by farmers, distribution costs for different regions, and maximum allowable profit margins. This uniform pricing approach is essential to ensure equitable pricing across the nation, mitigating unwarranted competitive price hikes. Furthermore, Ethereum introduces a layer of pseudo-anonymity, wherein transactions are conducted using unique addresses rather than personal names. This approach enables competitors to engage in buying and selling without unnecessarily escalating competition. However, should conflicts arise, the system retains the capability to access the entire transaction history, enabling the identification of discrepancies and the source of any issues.

The implementation of this system would be a localized endeavor, with each district and division overseen by the government. Proper training and guidance for stakeholders would be essential, and they would only require suitable hardware and software to access the network. Given the increasing accessibility of such technology, particularly with the prevalence of mobile devices, the system can be tailored for handheld mobile access. Concerning the costs associated with gas fees and validation in a private Ethereum network, these expenses can be customized to align with the specific requirements of users. Consequently, the associated costs would be significantly lower than the exorbitant price increases witnessed in recent times. It is noteworthy that the Bangladeshi government is actively working to enhance the country's digital capabilities, with a strategic goal of becoming blockchain-enabled by 2030, as outlined in the National Blockchain Strategy 2020. This aligns this type of system setup with the nation's broader objectives and aspirations.

### 4.5 Use case analysis

**Fig. 10:**
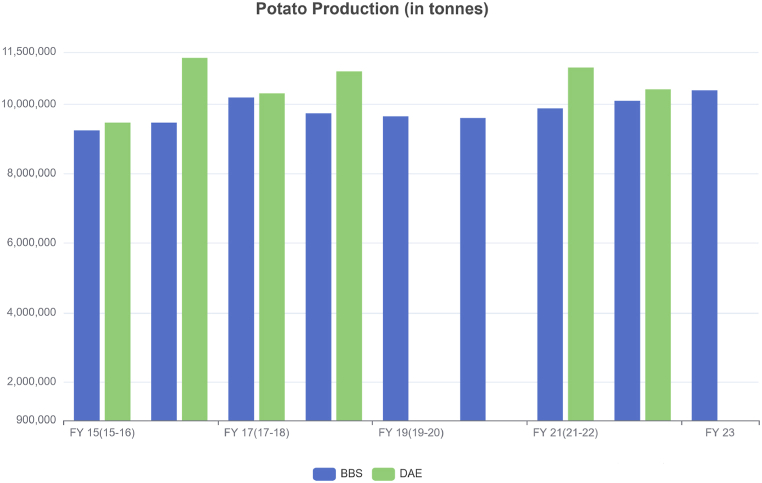
Potato production statistics from Fiscal Year (FY) 2015–2023 according to BBS and DAE.

**Fig. 11:**
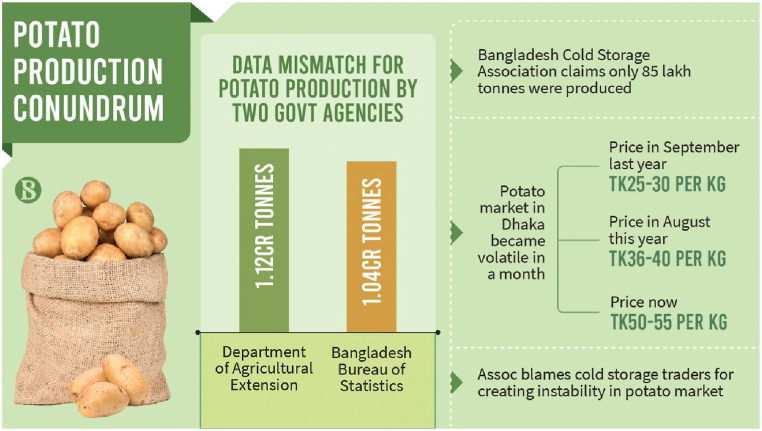
Potato production and price hike (Source: The Business Standard).

The Bangladesh Bureau of Statistics (BBS), the Department of Agricultural Extension (DAE), and the Trading Corporation of Bangladesh (TCB) are key government organizations in Bangladesh that offer valuable data related to agricultural statistics [[27], [28], [29]]. Fig. 10, Fig. 11 highlight that even government agencies struggle to monitor the supply chain, evident in the conflicting data they report regarding potato production.

Problem Description: Potatoes are a significant agricultural crop in Bangladesh, ranking as one of the primary staples following rice and wheat. Over the recent years, the country has consistently produced an average of 10 million metric tons of potatoes [30]. The Prothom Alo's report indicates that the current potato supply in the country's cold storage is sufficient until the second week of December. Additionally, new potatoes will become available in the market, ensuring there is no risk of a shortage. According to the Bangladesh Cold Storage Association (BCSA), the country produced 8.5 million metric tons of potatoes during the fiscal year 2022-23. In contrast, the Department of Agriculture Extension (DAE) reports a higher figure of 11.2 million tons. Notably, the demand for potatoes in the country is 9 million tons, affirming that there is an ample potato supply. During a recent press conference, cold storage owners raised concerns that certain unscrupulous traders are artificially inflating potato prices through manipulation. In just one week, prices have risen by BDT 5 (equivalent to USD 0.046) per kilogram, reaching a maximum of BDT 45 (about USD 0.41) in Dhaka's markets. This increase is attributed to a shortage in supply compared to the high demand. The current price is notably 57 per cent higher than it was a year ago. The upward trend in prices is a result of a deliberate slowdown by both traders and large-scale farmers who store their potatoes in cold storage facilities to release their stocks gradually during the off-season. This situation has been confirmed by industry stakeholders. Tajul Islam Patwary, director of field services at the Department of Agriculture: “This year's production is a record high in Bangladesh's history. Currently, there is no scarcity of potatoes in the country.” He attributed the price increase to insufficient market oversight by relevant authorities, including the Directorate of National Consumers' Rights Protection (DNCRP). Mostafa Azad Chowdhury, president of the BCSA: “We do not agree with the government data.” Around 20 per cent of the cold storage space owned by BSCA members has not been put to use. Mostafa Azad Chowdhury: “If the potato production is high, where are these surplus potatoes?” According to him, traders are deliberately releasing their stocks at a slow pace because they are aware that farmers have already depleted their stockpiles. (Source: The Daily Star)

Based on the provided description, we can make the following observations.

• Despite an ample supply of potatoes, prices are still surging.
• The current system lacks transparency and suffers from significant disparities in information between government entities and other stakeholders.
• In this situation, it is the farmers and consumers who are being deprived of their fundamental rights, while unscrupulous businesspersons are reaping the profits.

There is a visible asymmetry between production and prices. While government reports may capture some anomalies in pricing, the prices of essential commodities continue to increase without any apparent justification [31,32]. Therefore, the best solution to this problem is to create a system that is transparent, traceable, fraud-intolerant, trustworthy, highly immutable, and accountable. Blockchain technology provides all these solutions in one package. Moreover, a private Ethereum network offers greater customization to user needs and is more affordable. With such implementation, businesses can save billions of dollars while maintaining integrity. Established organizations such as Walmart and IBM Food Trust are leveraging blockchain technology to establish secure and reliable supply chain systems [33,34].

When implementing the proposed system, this is how the potato supply chain would operate.

Step 1 An administrative panel will be established with the involvement of relevant government officials, such as the local agriculture officer. This individual should possess a deep understanding of the local supply chain and its stakeholders, including farmers and other intermediaries.

Step 2 Subsequently, the admin panel will deploy smart contracts that encapsulate the rules and regulations governing each transaction within the system onto the private Ethereum network.

Step 3 Next, stakeholders will be integrated into the system, each assigned a unique Ethereum address by the admin or administrators. Each user must have their distinct Ethereum address, which they will use for conducting transactions within the network. Proper identification and verification for each user are mandatory.

Step 4 Farmers will be responsible for providing comprehensive details about their harvest's lifecycle. This includes information about the sowing and harvesting times, fertilizer usage, the irrigation system, temperature, pesticide types and quantities, and all relevant costs supported by evidence. The use of sensors and drones can be considered for monitoring and data collection. At each stage, the product's status should be updated. Large data files will be stored on IPFS, with links provided alongside other data on the blockchain.

Step 5 As the product's status changes to ‘harvested,’ an event will be triggered within the system to inform all stakeholders about the product's availability, along with the expected price set by the farmer (within government-defined price limits).

Step 6 If a buyer has sufficient credit, they can purchase the product. Each transaction will undergo validation through the PoA consensus mechanism and will be recorded in the blockchain. Over time, as transaction history grows, it will also be stored in IPFS.

Step 7 The product will be shipped to the buyer through distributors, with each step of the process documented in the blockchain.

Step 8 Upon receiving the product, the buyer will confirm receipt and provide evidence. If any issues are identified with the received product, they will report them with supporting evidence.

Outcomes: The proposed system within Bangladesh's potato supply chain offers the following advantages.

• Transparency: All transactions are visible to network members, ensuring complete openness.
• Traceability: Each transaction can be traced back to its respective Ethereum addresses, facilitating tracking.
• Fraud Prevention: Attempts to manipulate data for fraudulent transactions are deterred since all network members possess a copy of the blockchain.
• Security: Smart contract protocols automatically halt illegal transactions and generate alerts for address identification by emitting relevant events.
• Data Protection: Blockchain employs cryptographic methods to safeguard data and transactions, permitting authorized actions only.
• Transaction speed: Private Ethereum networks are known for their faster transaction processing. The utilization of the PoA (Proof of Authority) consensus mechanism further enhances transaction speed.
• Cost Efficiency: The system setup costs (software, hardware, network) and transaction expenses (gas fees) are lower compared to the public Ethereum network and can be adjusted to specific needs. This cost is substantially less than the financial losses incurred due to corruption in the current system.
• Price Reduction: Identifying the underlying issues within the potato supply chain at their source enables the elimination of problems, ultimately leading to a reduction in unjustified price increases.

The Bangladeshi government's attempt to regulate market prices and enforce adherence to the government-set rates involves consumer protection agents conducting widespread inspections of markets and stores across the nation. However, it's challenging to effectively control such a dysfunctional system using this approach. With the proposed system, these agents would have the capability to comprehensively monitor the entire supply chain and receive immediate alerts whenever any irregularities occur.

Other use cases: This system can be implemented in virtually any agricultural or food supply chain with minimal or no adjustments while retaining the same fundamental steps.

From the use case discussed above, it is evident that systems of this type have the capacity to revolutionize existing systems. The goal of the study, as suggested by the title, was to identify the origin of price hikes to eradicate the problem at its core. The proposed system can track and trace each transaction back to its origin, enabling the detection of the provenance of price hikes. Additionally, if an unscrupulous person attempts to sabotage the system by performing illegal transactions, the system will automatically halt those transactions. Due to the inherent qualities of blockchain, it is, in reality, very resource-intensive and nearly impossible to alter previous data or transaction history. This is because the data is hashed and distributed among all stakeholders in the system. This fact was further substantiated during our experimentation with a demo blockchain-based supply chain system, utilizing the Ganache and Truffle framework.

Now, when considering whether the system can truly solve the issue of price hikes, the answer is not straightforward. Our system can certainly detect price hikes but some other factors must be taken into account to make the system truly functional. First of all, stakeholders must understand that the system isn't against anyone but is for them. To encourage stakeholders to adopt these systems, various policies with incentives can be implemented.

• Financial incentives: Financial incentives such as grants and dedicated funds should be offered to promote the adoption of blockchain-based systems. Another critical aspect is the provision of aid or assistance, such as subsidies for product prices, which governments or operating authorities should provide. Additionally, reducing taxes on tools for agriculture and agri-food products in blockchain-based systems might further support this initiative. These incentives can encourage everyone to join the system. Proper efforts should be made to make people aware of the system's capabilities. The money saved by the proposed system will be significantly higher than the current losses incurred by the faulty system.
• Promoting Transparency and Trust: Transparency and traceability ensure that everyone receives their fair share. In the current system, the two most affected parties, the farmers and the customers, will gain a clear view of the product life cycle. For farmers, understanding the product life cycle is crucial to being aware of any price discrimination. On the other hand, customers can gain a clear idea of the product's origin and price. While the suggested system recommends removing unnecessary middlemen, there will inevitably be a few necessary intermediaries. To encourage them to use the system, they must understand that they can also benefit from it. However, their profit should be within a legitimate and justified limit agreed upon by the stakeholders, considering factors like production and distribution costs. Additionally, they can gain insight into the demand and supply ratio, helping them decide where to invest further.
• Training programs and workshops: To build the necessary skill set, training programs and workshops should be provided for public officials and relevant stakeholders. The government and technology providers can contribute to this effort. To demonstrate the potential of such projects in the real world, the government can allocate funds for trial or demonstration programs.
• Regulatory Support and Security: Local public officials can be added as admins with the authority to add and onboard other stakeholders. They can also educate these stakeholders on the system's rules and regulations. As the system focuses on the agri-food supply chain where product prices need to be within a specified range (which may vary according to different production techniques and related costs), competitors can profit within the predetermined range set by the stakeholders. Furthermore, Ethereum maintains pseudo-anonymity, resolving competition issues. However, whenever an illegal transaction occurs (according to the rules set by smart contracts), it will be halted and traced back to the origin, and the entity can be identified by its Ethereum address (as well as by the operators or admins). The system admins or operators should regularly monitor the system.

The system will unquestionably enhance the existing, highly complex, and unstructured system. For the system to operate successfully on a national or international scale, government support is crucial, not only at the central level but, most importantly, at the local level. Local governments must take the initiative to connect with local producers and farmers. It is essential for the general public to fully comprehend the structure, rules, benefits, and reinforcements of the existing system to facilitate its use. While it might be initially challenging to educate people in less developed countries or less developed parts of developed countries, it is achievable with sufficient effort and dedication from governments and the honest, marginalized parties that have suffered for years under the unjust system. Many countries are realizing the potential of blockchain technology and eagerly adopting it as a solution. Specifically, Bangladesh, on the verge of becoming a smart Bangladesh, can economically benefit from the revolution of using blockchain technology in supply chains. Furthermore, blockchain can reduce price asymmetry, resulting in synchronized value addition in the overall supply chain process. The market failures that have persisted for years are not mere assumptions; they are harsh truths that need to be addressed and dealt with in the most efficient way possible.

### 4.6 Comparison with existing systems

Table 3 presents a comprehensive assessment of the proposed system in contrast to the existing centralized system. This evaluation is founded on the issues addressed in the paper and the corresponding solutions. The fundamental criteria for this comparative analysis encompass transparency, traceability, reliability, cost-effectiveness, transaction speed, and security.

**Table 3:** Centralized system Vs Proposed system.

Parameters	Traditional System	Our Solution
Transparent	No	Yes
Traceable	No	Yes
Reliable	No	Yes
Cost-efficient	No	Yes
Fast	No	Yes
Secure	No	Yes

In Table 4, a comparative analysis is conducted to juxtapose this study with other relevant research efforts. This

**Table 4:** Comparison with existing works.

Parameters	Ekawati et al. [17]	Mukhtar et al. [18]	Salah et al. [35]	Mao et al. [36]	PS
Government Panel	No	No	No	No	Yes
Cost Analysis	No	No	No	No	Yes
Implementation and Testing	Yes	Yes	No	Yes	Yes
Security analysis	No	No	No	No	Yes
Vulnerability analysis	No	No	No	No	Yes
Proof of Concept	Yes	Yes	Yes	No	Yes

Comparison is based on a set of pertinent parameters that such systems typically encompass. Here PS stands for the ‘Proposed System’.

## 5 Conclusion

The purpose of the study was to use a private Ethereum blockchain network to identify the source of price hikes in the agri-food supply chain. To do that, we concentrated on the current agri-food supply chains and discovered that their design was largely to blame for undetected price increases. In order to track and trace a product's life cycle across the system, we presented a comprehensive (farmer-to-consumer) blockchain-based solution. This kind of scheme can eliminate needless middlemen while also assisting farmers and consumers to receive what they are due. Such systems can also benefit the government because they can identify who is to blame for price increases. Because the system is decentralized and immutable, both the government and the general public may trust its data. The system regulations were developed as smart contracts and implemented on a local blockchain. By creating a prototype dApp, we also showed the smart contracts in operation. Our approach can also guarantee food safety by identifying the cause of food contamination. The technique can be used to cut down on food waste as well. Finally, we presented the security and vulnerability study that demonstrated how dependable and safe our solution is. Since there are other elements at play, this kind of approach can't completely eliminate price increases, but it can assist in identifying and eliminating the major cause. The overall analysis demonstrates that the suggested blockchain-based system can outperform the conventional approach in terms of security and transparency. The proposed system is capable of carrying out the tasks required to accomplish our aim.

## Future scope

In this research, we introduced a conceptual model and developed a demonstration using the Truffle framework and a local Ganache blockchain. During this demonstration, we conducted transactions between stakeholders using experiment-specific Ethereum addresses, following the rules set by the smart contract. Any deviations from the system's expected behaviour were effectively identified and addressed by the system. The effectiveness of a technology is ultimately determined by its problem-solving capabilities. With the theoretical validation of our approach, our next step is to transition from theory to practice by transforming it into a real-world application. This practical application aims to address the issues elaborated upon in this paper. But to make our system more effective at serving as both an incentive and a guide for the stakeholders, we plan to add a blockchain-based reputation or review system to it. Additionally, we intend to create a mobile-based solution.

## Data availability statement

Data will be made available on request.

## CRediT authorship contribution statement

**Maksuda Haider Sayma:** Writing – review & editing, Writing – original draft, Visualization, Validation, Software, Resources, Methodology, Investigation, Formal analysis, Data curation, Conceptualization. **Md Rakib Hasan:** Writing – review & editing, Writing – original draft, Validation, Supervision, Project administration, Investigation, Formal analysis, Conceptualization. **Mahmuda Khatun:** Writing – review & editing, Investigation, Formal analysis. **Alimul Rajee:** Resources, Formal analysis. **Amena Begum:** Software, Investigation.

## Declaration of competing interest

The authors declare that they have no known competing financial interests or personal relationships that could have appeared to influence the work reported in this paper.
